# Nanomaterials for Drug Delivery to the Central Nervous System

**DOI:** 10.3390/nano9030371

**Published:** 2019-03-05

**Authors:** Daniel Mihai Teleanu, Irina Negut, Valentina Grumezescu, Alexandru Mihai Grumezescu, Raluca Ioana Teleanu

**Affiliations:** 1 Emergency University Hospital, Bucharest, Romania, “Carol Davila” University of Medicine and Pharmacy, 050474 Bucharest, Romania; daniel.teleanu@umfcd.ro; 2 National Institute for Lasers, Plasma, and Radiation Physics, RO-77125 Magurele, Romania; negut.irina@inflpr.ro (I.N.); valentina_grumezescu@yahoo.com (V.G.); 3 Faculty of Physics, University of Bucharest, 077125 Magurele, Romania; 4 Faculty of Applied Chemistry and Materials Science, Politehnica University of Bucharest, 011061 Bucharest, Romania; 5 ICUB—Research Institute of University of Bucharest, University of Bucharest, 36-46 M. Kogalniceanu Blvd., 050107 Bucharest, Romania; 6 “Victor Gomoiu” Clinical Children’s Hospital, “Carol Davila” University of Medicine and Pharmacy, 050474 Bucharest, Romania; raluca.teleanu@umfcd.ro

**Keywords:** neurodegenerative disease, blood brain barrier, nanoparticles, Alzheimer’s disease, Parkinson’s disease

## Abstract

The intricate microstructure of the blood-brain barrier (BBB) is responsible for the accurate intrinsic regulation of the central nervous system (CNS), in terms of neuronal pathophysiological phenomena. Any disruption to the BBB can be associated with genetic defects triggering or with local antigenic invasion (either neurotoxic blood-derived metabolites and residues or microbial pathogens). Such events can be further related to systemic inflammatory or immune disorders, which can subsequently initiate several neurodegenerative pathways. Any degenerative process related to the CNS results in progressive and yet incurable impairment of neuronal cells. Since these particular neurons are mostly scanty or incapable of self-repair and regeneration processes, there is tremendous worldwide interest in novel therapeutic strategies for such specific conditions. Alzheimer’s and Parkinson’s diseases (AD and PD, respectively) are conditions found worldwide, being considered the most rampant degenerative pathologies related to CNS. The current therapy of these conditions, including both clinical and experimental approaches, mainly enables symptom management and subsidiary neuronal protection and even less disease regression. Still, a thorough understanding of the BBB pathophysiology and an accurate molecular and sub-molecular management of AD and PD will provide beneficial support for more specific and selective therapy. Since nanotechnology-derived materials and devices proved attractive and efficient platforms for modern biomedicine (including detection, imaging, diagnosis, medication, restoration and regeneration), a particular approach for AD and PD management relies on nanoparticle-based therapy. In this paper we will discuss relevant aspects related to the BBB and its impact on drug-based treatment and emphasize that nanoparticles are suitable and versatile candidates for the development of novel and performance-enhanced nanopharmaceuticals for neurodegenerative conditions therapy.

## 1. Introduction

Nanotechnology represents a multidisciplinary field that covers the design, fabrication and functionality of materials and devices with dimensions in the nanometer (nm) domain. The National Nanotechnology Initiative terms nanotechnology as the fabrication of materials in a scale between 1 to 100 nm in at least one dimension [1]. According to the National Institute of Health, nanomedicine is an attractive and challenging field of nanotechnology-derived applications, in terms of novel, specific and selective medical products intended for treatment and therapy areas such as imaging, diagnosis, drug and cell therapy, tissue regeneration, etc. [1,2]. Nanotechnology’s innovations, known as nanoparticles (NPs), have been applied in medicine for the diagnostic, treatment and/or prevention of human diseases, since NPs’ dimensions are comparable to those of biomolecules, such as proteins (1–20 nm), DNA (with a diameter of ~2 nm), hemoglobin (∼5 nm), viruses (∼20 nm), cell membranes (∼6–10 nm) [3]. In nanomedicine, it has been proposed to extend the range of nanoscale materials and devices to 1000 nm [4]. The latest market reports anticipate that the use of nanotechnology in medicine could extent the financial implications to $528 billion by 2019 and will continue to grow substantially in the years to come [5].

Nanomaterials can enter the body by many paths, such as the respiratory route, skin, digestive canal and drug injection, followed by their transportation into organs and succeeding their biological effects (including inflammatory responses, oxidative stress, cellular apoptosis and DNA damage). NPs became an essential subject of drug delivery research, because they can load and deliver an impressive range of medications to almost any organ/area of the body, providing targeted, controlled and sustained therapeutic effects [6]. Drugs or other bioactive molecules can be dissolved into NPs, or they can be trapped, encapsulated and/or adsorbed or attached. Many studies reported the successful delivery of hydrophilic and hydrophobic drugs, proteins, biological macromolecules and even vaccines by using NPs as carriers [7]. NPs have a further advantage over larger microparticles, given their suitability for intravenous administration and their tremendous potential for controlled drug release and site-specific drug targeting [8].

Neurological maladies consist of a comprehensive variety of ailments that have a major impact on a high percentage of the global population and, according to many studies, are estimated to grow with the ageing of the population [9]. The latest data published by the World Health Organization (WHO) make known that stroke is the second chief cause of death worldwide, while dementia is the seventh. Among the broad category of dementia types, Alzheimer’s disease is the main condition that impairs human brain [10]. Moreover, in the last decades, the autism, traumatic brain injury, stroke, schizophrenia, Parkinson’s and Alzheimer’s diseases (apart from epilepsy, depression and chronic pain) are merely a few from the great number of central nervous system (CNS) pathologies that benefited from advancements of the modern neurology research. Even though the genetic root for several of these disorders is known, there is still no cure or even a fully functional treatment to slow their progression [11]. Thus, these conditions denote an outsized area of unmet medical necessity and result in a major socio-economic problems. Besides, the presence of the blood–brain barrier (BBB) constrains the entrance of brain-targeting drugs into the brain; around 98% of medications do not enter the BBB [12,13]. Therefore, the need for new therapeutic approaches of CNS diseases and the limitations caused by the obstruction imposed by the BBB are promoting the advancement of nanotechnology in targeted drug delivery. For example, NPs can be suitable drug carriers to the CNS thanks to their physical (mostly, size-related features) and chemical properties. When manipulated in a precise way, NPs can overcome the BBB [14] proficiently.

Another priority in finding a cure for neurodegenerative diseases is investigating pathophysiological mechanisms of underlying brain disorders. For example, the pathological processes underlying Alzheimer’s predominantly involve the temporal evolution of Alzheimer’s biomarkers in relation to each other and to the beginning and progression of clinical symptoms. Other biomarkers are the intraneuronal establishment of abnormal proteins and extracellular deposition of specific proteins [15]. In this respect, one modulates the brain activity towards a healthy physiological function.

The present paper focuses mainly on the characteristics of the BBB and its permeability, passage strategies for transporting drugs across the BBB, and the latest strategies regarding the use of many complexes to customize NPs to enter the brain (in an attempt to provide an appropriate therapeutic approach for different CNS diseases).

## 2. Mechanisms to Surpass the Blood–Brain Barrier (BBB)

Neurons, which are post-mitotic cells, are exceptionally sensible to both internal and external turbulences. This is why all molecular species that come in contact with neurons must be extremely well controlled. The CNS has established a succession of barriers in order to shield itself from intrusive chemicals and pathogens. Regrettably, a high number of pharmacologically active substances that could target CNS disorders are not able to employ their activity due to CNS’ formidable barriers: BBB, blood–cerebrospinal fluid barrier, cerebrospinal fluid–brain barrier, and some dedicated barriers like blood–tumor barrier (in the event of brain tumors) [16].

### 2.1. BBB—General Concept and Mechanisms of Passage and Transport

The CNS contains blood capillaries that are architecturally unlike those that originate in other tissues. The differences result in the formation of a specific permeability barrier amidst the blood inside brain capillaries and the extracellular fluid in the brain tissue. In particular, the capillaries of brain and spinal cord are characterized by the absence of small pores that otherwise have the role to enable the transfer of solutes from the circulation into different organs [17]. The capillaries are packed with a layer of distinctive endothelial cells that are wrapped with tight junctions (TJ), the role of which is to limit molecules and ions to pass through intercellular spaces. In other organs (such as skin, bladder, colon and lungs), a similar tight epithelium can also be found [18].

From the anatomical point of view, the BBB is composed from the following parts: endothelial cells (also known as brain microvascular endothelial cells) united by tight junctions (TJ), basal lamina, pericytes embedded in basal lamina, astrocytes with their projections—endfeet—touching the abluminal side of brain vessels and neurons [19].

The normal function and maturation of endothelial cells is mainly dependent on the junctional complexes, namely TJ and adherens junctions (AJ) protein expression, upregulated by intercellular interfaces of brain stromal cells and endothelial cells [20]. TJ are established more apical than AJ, they constitute a limit for the passage of polar solutes by paracellular ways and are made by occludin, claudins, and junctional adhesion molecules [21]. The occurrence of these proteins is imperative for BBB functioning [22]. Conversely, cadherin family proteins form AJ are principally in charge for the structural sustenance and tightness of the BBB, which can be evaluated as the electrical resistance through endothelia (transendothelial resistance).

The pericyte cells are contractile cells that cover ~20% of endothelial cells’ external surface and can be credited for both the vascular smooth muscle cell lineage and for adjusting the microcirculatory blood flow in the brain capillary (by contraction and relaxation) [23,24]. They are located in close vicinity to astrocytes and neurons [25].

The astrocytes unite neurons and the brain capillary. They keep BBB functions by delivering nutrients to neurons and safeguard the brain from oxidative stress and metal toxicity [26]. Furthermore, in vitro and in vivo investigations confirmed the significance of astrocytes in BBB integrity [27]. The basement membrane comprises endothelial cells and pericytes and encloses extracellular matrix proteins, like heparan sulfate proteoglycans, type IV collagen, etc. [28]. Moreover, these endothelial cells are enveloped by a self-secreted structural assembly known as basal lamina that is composed of fibronectin, heparan sulfate, laminin and type IV collagen [29].

The brain microvascular endothelial cells are surrounded by pericyte contractile cells that prevent the barrier damage caused by immune cells. Pericytes cells further contribute to the BBB efficiency with their phagocytic capacity and act by reducing proteins levels associated with cell permeability [30,31]. Another key assembly involved in the inhibition of solute transport is the basal membrane (constituted by collagen, fibronectin and laminin). Furthermore, astrocytes’ endfeet surround endothelial cells and contribute to the reduction of BBB permeability; astrocytes also provide biochemical support to endothelial cells [32]. Close to endothelial cells, neuronal endings are also present.

The brain uptake of a certain substance is dependent on several aspects, like the affinity of a substrate for a particular transference system and its molecular weight. Furthermore, solutes crossing the cell membrane are susceptible to action of enzymes that reside in endothelial cells, which recognize and quickly destroy most of peptides (even naturally occurring neuropeptides). Also, endothelial cells of the brain capillary contain high concentrations of drug efflux transporter proteins (such as P-glycoprotein, multidrug resistance-associated proteins and breast cancer resistance protein), which restricts the infiltration/diffusion of a variety of healing agents. Consequently, most of the endogenous components and even nutrients are transported into the brain by diverse transporters expressed by the BBB.

The insoluble lipids and large hydrophilic molecules’ infiltration/diffusion into the brain is mostly hindered and the metabolic process need to be transcellularly exchanged across the BBB by active transport by means of specific proteins [33].

The BBB is also characterized as a metabolic blockade, as it contains enzymes that are proficient in metabolizing and disabling drugs, toxic and neuroactive complexes [34]. The tight junctions between endothelial cells practically seal the BBB and consequently restrain the admission of systemically circulating endo-/exogenous composites and hydrophilic molecules into the CNS [35].

**Figure 1:**
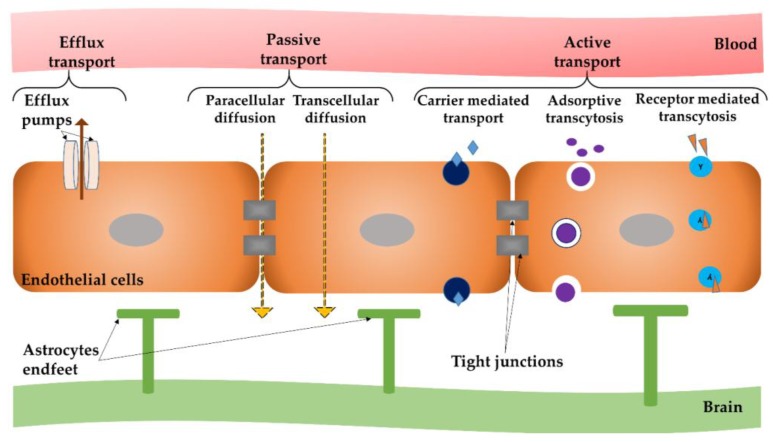
Different modes of transport across the blood–brain barrier (BBB).

Although there is a variety of molecular transport routes that can cross endothelial and/or epithelial barriers in other organs, brain capillaries possess a reduced number of “windows” between neighboring endothelial cells that permit molecules, along with cells, to move without difficulty across the endothelium. Therefore, the solutes’ stream across the BBB is more controlled than in the case of general capillaries; larger molecules are disallowed from passing the barrier and carried into and out of the brain tissue, without being recognized by specific proteins [36]. Depending on both the solutes form, including their physical and chemical properties together with the biological structures included in the blood vessel wall, solute molecules infiltrate the barrier through a specific route and by particular mechanisms [37]. There are several routes of molecular passage across the BBB, as outlined in Figure 1.

Molecules absorption across the BBB occurs by means of two mechanisms: passive and active transport [38].

Passive transport, also known as passive diffusion, comprises the following non-energetic transportation pathways: (i) paracellular diffusion—the mechanism of passage between endothelial cells of hydrophilic compounds; and (ii) transcellular diffusion—the passage through endothelial cells, which is used by small lipophilic molecules to reach inside the brain parenchyma. Passive transport enables the system to reach its inner entropy, by considering biologically occurring gradients.

The degree by which a substance can undergo passive diffusion depends on several intrinsic factors, such as pharmacokinetics, hydrogen bonding and charge. This process can be evaluated by the octanol/water partition coefficient, possible values of which should be between 10:1 and 100:1 to successfully provide passive transport [39]. Examples of marketed drugs that access the brain by this pathway are steroids and diphenhydramine [40,41]. The balance between paracellular–transcellular transports is pivotal for expressing the grade of permeability in the BBB [42]. Since lipid solubility is a key factor in passive transport into the BBB, this method involves the chemical transformation of water-soluble molecules into lipid-soluble ones proficient in crossing the BBB. This is achieved by the addition of lipid or functional groups to the polar ends of drug molecules [43].

The paracellular pathway partially influences the substances entering into to the brain. This is usually the case of molecules that have long half-lives, small distribution volumes and strong effects on CNS. Erythropoietin and antibodies are some examples [44].

The transcellular route takes place in many cases with passive diffusion of liposoluble molecules, water and some gases (e.g., O_2_, CO_2_) [45]. Nevertheless, lipophilicity cannot solely regulate the membrane absorbency of a molecule. The limit for the passage through the BBB, regardless the lipophilicity, is that molecules should have a molecular weight of ~(400–500) Da [46]. It was found that some lipophilic tranquilizers (such as benzodiazepines) can quickly travel across the BBB [47], opposed to other lipophilic molecules such as immunosuppressants (like cyclosporine A) [48].

Active transport enables the movement of substances (such as nutrients, ions and endogenous substances) into the brain alongside their gradient, but in an energy-dependent mode. The active transport comprises energy-dependent mechanisms that use primary or secondary cellular energy to overcome biological gradients (such as concentration or electrochemical gradients) and other biological resistance. This pathway is utilized by many therapeutic molecules (such as opioid analgesics, cardiac glycosides and calcium channel blockers) to gain access to the CNS [49]. This type of transport includes: (i) carrier mediated transcytosis that is suitable for relatively small molecules; (ii) absorptive mediated transcytosis for positively-charged peptides; and (iii) receptor-mediated transcytosis.

Receptor-mediated transcytosis is a key transportation path for endogenous peptides, like insulin, transferrin, insulin-like growth factor and nicotinic acetylcholine receptor [50]. These receptors can specifically bind to conforming ligands and activate internalization into cells. It is a specific process and it uptakes macromolecules existing on the luminal side of brain endothelial cells and transports them to the brain with the receptor reprocessed back to the luminal membrane [51].

Absorptive-mediated transcytosis does not implicate plasma membrane receptors. This passage mode can be triggered by means of electrostatic interfaces between polycationic molecules and the plasma membrane (that possesses a negative charge). The negatively-charged surface of endothelia provides specific interactions with positively-charged blood proteins, resulting in a non-selective transport way across BBB. Consequently, owing to the electrostatic interaction amid negatively-charged membranes, the cationic therapeutic complex takes the absorptive mediated transcytosis to enter the CNS. This path can facilitate albumin transport; for example cationized albumin was used as a targeted drug delivery approach in investigational models of neurodegeneration [52].

Carrier-mediated transport represents the path by which nutrients can be delivered into the brain by capillary endothelial luminal and the abluminal membranes [53]. The abluminal membrane of endothelial cells is associated with the brain extracellular fluid, whereas the luminal membrane is associated with the blood component [54]. There are specialized transporters which provide the passage of amino acids across the BBB. For example, glucose transporter (GLUT-1) facilitates the crossing of glucose to the brain [55]. Valine, histidine, methionine and tyrosine are energy-dependent, and carried by System-L transporter. A classic example of drug that enters the CNS by this mechanism is the L-DOPA or Levodopa anti-Parkinson drug, which operates with large amino acid transporter 1 (LAT-1) [56]. The same mechanism elucidates how glucose, vitamins and some peptides can cross the brain at a faster rate than would be expected, centered on both their physical and chemical characteristics [57].

Efflux mechanisms also exist in the BBB. The most well-known is the P-glycoprotein (Pgp) mechanism that has a predisposition for pumping out undesirable complexes, such as anticancer drugs and antibiotics [30,58].

Nevertheless, some regions of the brain do not possess the BBB and are acknowledged as circumventricular organs (localized next to ventricles). These regions are vascularized, but their capillaries’ surface area is inferior, as paralleled to areas where BBB is present, causing the dispersion ratio of molecules to CNS [59].

### 2.2. Access of Nanoparticles to the Central Nervous System

NPs are extraordinary versatile and multifunctional structures that can be applied for brain drug delivery, thanks to the impressive opportunity to tune their engineering process, in terms of shape, size, hydrophobicity, surface chemistry and charge, etc. NPs can be modified to include some or all of the subsequent features: (i) biocompatibility; (ii) reduced toxicity; (iii) ability to bind and transport multiple loads; (iv) shield therapeutics from in vivo degradation; (v) control of therapeutics release for prolonged periods of time; and (vi) navigate the BBB. A complete control over these features can enhance BBB penetration efficacy [60].

The current strategies to accomplish drugs/NPs infiltration into the CNS comprise:

(i) non-invasive methods based on drugs modification to increase BBB permeability, such as intranasal delivery [61]; and
(ii) invasive techniques that necessitate direct intraventricular or intracerebral injection/implantation, infusion [62]; or
(iii) provisional disruption of the BBB [63].

The disruption of the BBB is an approach extensively used to increase drug delivery efficiency to the brain. For example, the BBB disruption could be made by osmotic opening using mannitol (for the treatment of certain CNS cancers) [64] and by using ultrasound to open the transient pore in the BBB [65]. Recent research revealed that pathological BBB disruption occurred in the case of Alzheimer’s and multiple sclerosis, and resulted in no intensification in permeability for small therapeutic molecules (<1000 Da) [66].

The intrinsic physical and chemical properties of NPs decide the route and mechanisms of crossing the BBB. Some studies suggest that NPs functionalized with an appropriate ligand are capable of crossing the BBB without obvious damage and can be used to transport and distribute drugs and/or genetic material into the lesioned brain [67,68]. Taking into account the previously discussed transport mechanisms that are described in Figure 1, NPs can:

(i) open TJ or cause local toxic effects, which can result in an enhanced permeabilization of the BBB, letting drugs or drug-conjugated NPs infiltrate into the CNS [69];
(ii) pass through endothelial cells by transcytosis mechanism [70];
(iii) be transported into endothelial cells by endocytosis, followed by their intracellular cargo release and their endothelial abluminal exocytosis [71]; or
(iv) cross the BBB by a combination of the previously described mechanisms.

Various research studies proved that transcytosis and endocytosis mechanisms are the main routes of transport for NPs. By understanding the receptor-mediated transcytosis and the adsorptive-mediated transcytosis mechanisms, as well as by considering the outcomes related to the passive diffusion process, the emerging niche research area of modern neuropharmaceuticals with specific physicochemical properties and biofunctional features can enable them to undertake transcytosis and cross the BBB, as well as to provide a patient-oriented therapy [72].

Passive transport can be enabled by boosting of a drug’s plasma concentration. This results in a superior gradient at the BBB and consequently a growth of the drug quantity that is inflowing the CNS. Furthermore, degradation products of NPs which present pro-adsorption properties, could increase the passive diffusion [73]. For example novel NPs comprised of emulsifying wax and Brij 78 was shown to have significant brain uptake. To target the brain in a specific manner, authors incorporated thiamine as a surface ligand on NPs. The assays performed to obtaine NPs brain uptake demonstrated that thiamine-coated NPs associated with the BBB thiamine transporter had an increased K_in_ between 45 and 120 s (thiamine coated NPs 9.8 ± 1.1 × 10^−3^ mL/s/g) versus uncoated NPs (7.0 ± 0.3 × 10^−3^ mL/s/g) [74]. Another recent study was designed to develop and optimize lazaroid loaded nano-structured lipid carriers (LAZ-NLCs) [75]. Their dimensions were chosen in the range of (150–200 nm) for enhancing lazaroid brain exposure and for bypassing the clearance mechanisms, resulting in a longer contact time with the BBB for passive diffusion. The optimal LAZ-NLCs increased the brain permeability by 2 times and residence by 1.5 times in the treatment of glioblastoma.

The receptor mediated transcytosis mechanism is based on the reciprocal action of NPs surface ligand and a specific receptor in the BBB. The postulated steps for BBB crossing entail the interaction of receptor-modified NPs, formation of endocytotic vesicles, transcytosis across endothelial cells of the BBB, followed by exocytosis of NPs. Actually, some research groups functionalized the surface of NPs-based drug transporters with ligands that promote their specific binding to receptors on the surface of brain endothelial cells. Specifically, NPs target transferrin [76] and lipoprotein receptors [77]. PEGylated immunoliposomes can enter the CNS arbitrated by an antibody (such as OX26) that links to the transferrin receptor, and transport their cargo into the brain without harming the BBB [78]. Accordingly, limitations of receptor-based methodologies are centered on the bond between the receptor and the ligand from NP surfaces, resulting in a low exocytosis rate. The limitations are emphasized in several publications [79]; it was found that a higher percentage of NPs reside in capillary endothelial cells compared to CNS parenchyma. Even though more receptors have been discovered, the differential expression, delivery and instruction of receptors at BBB in the diseased brain are not yet completely understood.

On the other hand, the modulation of drugs’ surface charge attracted significant attention regarding the stimulation of adsorptive-mediated transcytosis of neuropharmaceuticals across the BBB [80,81]. In a study, authors have “merged” several tactics in order to improve the brain uptake of NPs. The authors designed PEGylated chitosan (CS) NP grafted (CS-PEG-OX26)/not grafted with the targeting ligand OX26 (CS-PEG) [82] in order to: a) increase blood circulating time (with the help of PEG); b) undergo adsorptive-mediated transcytosis (owing to electrostatic interfaces between polycationic CS and negative charged endothelial cells’ membrane; c) undergo receptor-mediated transcytosis because of OX-26 mAb selectivity. The results obtained showed that NPs were frequently situated in the hippocampus and that these NPs are able to cross the BBB.

It was reported that small interfering RNA (siRNA)-mediated silencing of the P-gp gene responsible for efflux mechanisms is a viable strategy to progress drugs delivery to the brain [83,84]. Many research groups suggested nanosystems capable of carrying siRNA to the BBB; with the aim of further silencing the P-gp protein receptor, and temporarily making the BBB more permeable for P-gp substrates. For example, Malmo et al. explored the potential of siRNA-chitosan NPs in silencing P-gp in a BBB model. The results exhibited that P-gp silencing by chitosan siRNA NPs produced an improved delivery and efficacy of doxorubicin [85]. In another work, Gomes et al., designed a siRNA transporting nanosystem targeted against P-gp. In their study, polymeric poly(lactide-co-glicolide) (PLGA) NPs, with dimensions of ~115 nm in size, presented 50% siRNA association efficiency. The NPs surface was modified with a peptide binding to transferrin receptor (TfR), and their targeting ability against human brain endothelial cells was confirmed. The best functional nanosystem was PLGA and PLGA:PLGA-PEG-NH2 (95:5) was demonstrated to be harmless for human brain endothelial cells and without noteworthy cytotoxicity [86].

There are some parameters that can improve the effectiveness of NPs systemic circulation, BBB passage and drug delivery. Research studies revealed an opposite correlation between NPs size and BBB penetration [87]. More specific, research conducted on stroke, Alzheimer’s or animal models requires the use of NPs with diameters between (50–100) nm. Moreover, NPs morphology (e.g., spherical, cubic, rod-like) impacts their cellular uptake and subsequent body distribution [88].

The therapeutic approaches to disrupt the BBB and the suboptimal pharmacokinetic nature of many drugs can have indefinite consequences regarding the specificity and selectivity of the therapy performed. The weakness of these strategies relies on their selectivity absence, denoting that not only drugs get access the brain, but also other harmful substances (usually barred by the BBB) can get access to the CNS. Moreover, BBB disruption by drugs could result in a particular degree of barrier selectivity. Moreover, these methods may heighten the risk for cerebral infection, injury and toxicity, and often result in non-uniform drug distributions. As conventional drug delivery procedures have restricted entrance pathways to the brain, repeated systemic high doses are required, so that the drug can reach beneficial therapeutic levels in the lesioned CNS. This translates into risk damage due to the exposure of normal healthy tissues.

## 3. Current Research Advances in Nanoparticle-Based Treatment of Central Nervous System (CNS) Diseases

### 3.1. Nanoparticles for Alzheimer’s Disease

Alzheimer’s disease (AD), the representative and most widespread condition of chronic neurodegenerative pathologies, is currently an incurable disease. AD is the most prevalent type of dementia found in the elderly population, without an effective treatment or definite diagnosis. Besides the genetic implications related to the occurrence of such a condition, it is thought that AD is triggered by the buildup of amyloid-β (Aβ) peptides in the brain.

Amyloid-β peptide (Aβ), the sticky peptide, found in the brain plaques specific for AD, was first sequenced from meningeal blood vessels of AD and Downs syndrome patients [89]. Right away, the peptide was documented as the prominent element of senile (neuritic) plaques, specific for the brain tissue of AD patients [90]. This finding marked the beginning of modern research on AD. The neuropathology of AD is commonly linked to the subsequent cloning of the gene responsible for encoding the β-amyloid precursor protein (APP) and its localization to chromosome 21 [91,92], together with the earlier recognition of trisomy 21 (Downs syndrome) [93]. These discoveries are the starting point for the proposal that Aβ accumulation is the key occurrence in AD pathogenesis. Moreover, it was found that mutations of the APP gene (that cause hereditary cerebral hemorrhage with amyloidosis (Dutch type)) could have effects in Aβ deposition, mostly outside the brain parenchyma [94].

Brain accumulation of amyloid-β (Aβ) peptides, in the form of deposited plaques, represents one of the pathological pointers of AD [95]. It has been hypothesized that Aβ accumulation can be a consequence of a discrepancy between Aβ production and clearance; actually, Aβ clearance seems to be weakened in both early and late forms of AD and possibly contributes to the onset and progression of the disease [96]. In this respect, a novel therapeutic stratagem based on dropping levels of soluble Aβ assemblies in both the brain and the cerebral blood vessel, using the peripheral-sink effect, has been suggested [97]. β-amyloid represents a natural substrate of Pgp, and it is detached continuously from the CNS. Any mutation of the Pgp gene could lead to β -amyloid plaques accumulation, that is the main cause of the onset of Alzheimer’s disease [98].

Therefore, many strategies in combating this disease have been focused on either the prevention of or dissolving these peptides.

As the accumulation and/or the formation of Aβ plaques are some of the most prominent pathological hallmarks of AD, designing NPs- contrast agents that can selectively bind to Aβ, are br capable platforms for aiding the timely detection of AD. In this respect, Nasr et al. synthesized Sialic acid-coated bovine serum albumin (BSA) magnetic NPs which bind to Aβ deposits, with high selectivity [99]. The NP-BSAx-Sia could penetrate the BBB and allow for the detection of Aβ plaques using magnetic resonance imaging (MRI) in an AD mouse model without requiring of mannitol to open the BBB. Moreover, Aβ plaques perform as metal sinks causing Zn ions to have abnormally high concentrations with exhausted levels in the adjacent vicinity [100]. This Zn-ion sequestration by Aβ can lead to a diminished synapse density [101], and an amplified expression of pro-inflammatory cytokines. For that reason, local zinc deficit can cause microglia and astrocytes activation resulting in apoptosis and neuroinflammation [102,103]. Another research group [104] applied zinc-loaded NPs in wild-type and APP23 mice. The authors tested the effects of Zn-loaded NPs on plaque load, inflammation and synapse stability, as well as plaque amount and area, together with Zn concentration. Moreover, the activity, anxiety and cognition of mice was also verified

However, presently there are only four operative drugs that can be applied for the treatment of AD. They are from the class of cholinesterase inhibitors (donepezil, rivastigmine and galantamine) and the glutamate antagonist memantine [105]. But the existing drugs can only reduce the symptoms of AD without healing effects. Consequently, new treatments that will inhibit, delay or treat symptoms of AD are urgently needed.

Neuronal death and the improvement of AD patients’ cognition can be alleviated by using the memantine (MEM) drug. MEM drops the excess of glutamate that is responsible for neuronal death of AD patients [106]. In a recent report, physicochemically stable MEM–PEG–PLGA NPs were developed [107]. The NPs had a mean size smaller than200 nm (152.6 ± 0.5 nm), a monomodal size distribution (polydispersity index, PI < 0.1) and a negative surface charge of −22.4 mV. NPs were physicochemically characterized. It was found that the drug was dispersed inside the PLGA matrix. MEM–PEG–PLGA NPs were non-cytotoxic to brain cell lines (bEnd.3 and astrocytes) and were able to cross BBB both in vitro and in vivo. Behavioral tests on transgenic APPswe/PS1dE9 mice proved that MEM–PEG–PLGA NPs decreased memory impairment as compared to the free drug solution. Histological studies confirmed that MEM–PEG–PLGA NPs attenuated β-amyloid plaques and the corresponding inflammation of AD.

In another study, Tiwari et al. encapsulated curcumin (Cur)—the main pigment of turmeric—into highly lipophilic biodegradable PLGA (Cur-PLGA) NPs. It was showed that after intraperitoneal administration, in a rat model of Alzheimer’s disease, Cur-PLGA NPs stimulated adult hippocampal neurogenesis and liberated the animal from cognitive decline [108]. Fan et. al. synthesized a novel nanomaterial, Cur-loaded PLGA-PEG-B6 micelles (PLGA-PEG-B6/Cur), with PLGA-PEG in order to increase the bioavailability and B6 peptide to raise the BBB permeability of Cur. The aim of the study is to assess the efficacy of PLGA-PEG-B6/Cur nanomaterial for the treatment of AD. Authors investigated the drug-loading capacity, drug-release kinetics, blood compatibility, cell viability, together with the cellular uptake of PLGA-PEG-B6/Cur in vitro. In vivo tests on APP/PS1 transgenic mice showed an attenuation of memory loss and cognitive impairment [109]. Another possibility is given by targeting tacrine anti-AD drug in the brain, by means of polymeric NPs [110].

After many failures of Aβ-targeting drugs, there is keen interest in discovering beneficial potential tau-targeting. Neurofibrillary tangles, one more intracellular hallmark of AD, are composed of tau. Tau represents a microtubule-associated protein that is supplemented with axons. In some pathological conditions that occur independently of Aβ, the aggregation of tau will impair neuronal axons and, consequently, provoke neurodegeneration and cognitive deficits [111]. Moreover, tau is also implicated in the axonal transport of organelles (including mitochondria); the tau function loss causes mitochondrial dysfunction and oxidative stress [112,113] Tau stimulates microtubule polymerization and stabilization, and abilities are modulate by phosphorylation [114]. Thus, anomalous tau is significantly implicated in the pathogenesis and progression of AD, and novel approaches targeting tau pathology could be of great value for AD treatment.

Glat et al. engineered bioactive fibrin^377–395^ peptide intercalated into iron oxide NPs and, in effect, facilitated the inhibition of activated microglial cell-mediated tau tangle constitution in transgenic mice [115]. In a recent study, Chen et al. [116], constructed a methylene blue (MB, a tau aggregation inhibitor) loaded nanocomposite (CeNC/IONC/MSN-T807). The novel nanocostruct not only possessed a high linking affinity to hyperphosphorylated tau, but also impeded some of the most important pathways of tau-associated AD pathogenesis. The authors demonstrate that CeNC/IONC/MSN-T807 relieved AD symptoms by mitigating mitochondrial oxidative stress, suppressing tau hyperphosphorylation and protecting neuronal death both in vitro and in vivo. The memory deficits of AD rats are considerably alleviated upon treatment with MB-loaded CeNC/IONC/MSN-T807. Xu et al. [117] fabricated protoporphyrin IX (PX) modified oxidized mesoporous carbon (OMCN) nanospheres (PX@OMCN@PEG(OP)@RVGs) as AD multifunctional nanodrug with multiple target efficiency. The nanodrug was an efficient inhibitor of tau phosphorylation. In addition, the use of PX with focused ultrasound (US) triggered reactive oxygen species (ROS) production, that inhibited Aβ aggregation. This system increased the cognitive level of APP/PS1 transgenic (Tg) mice and achieved a dual-target inhibition of AD. In addition, the delivery of PX across the BBB in a safe and favorable manner, due to modification of the RVG peptide, was demonstrated both in vivo and in vitro. The photothermal effect of NPs enhanced the BBB permeability of PX@OP@RVGs under near-infrared irradiation. The presented results demonstrated the multifunctional and dual targeted treatment ability of PX@OP@RVGs for AD and crossing the BBB.

### 3.2. Nanoparticles for Parkinson Disease

Parkinson disease (PD) represents the second common neurodegenerative condition that has a negative impact on ~2–3% of the population aged ≥65 years. The neuronal loss in the black substance (*substantia nigra*) of the midbrain, which induces striatal dopamine deficiency and intracellular inclusions (that comprise aggregates of α-synuclein), represents the neuropathological hallmark of Parkinson disease.

The clinical diagnosis of PD is centered on identifying some mixtures of cardinal motor signs of bradykinesia, rigidity, tremor, and postural instability, but there are few efforts to develop a clear diagnostic criteria.

Most of James Parkinson’s clinical observations (almost 200 years ago) from the seminal essay on ‘the shaking palsy’, are still available nowadays. Aside from the general perception that Parkinson disease represents only a condition of impaired movement, it became obvious that a plethora of non-motor aspects like cognitive diminishment, autonomic dysfunction, sleep disorders, or even depression and hyposmia, are characteristics of this devastating disease. In this respect, there has been a remarkable evolution in understanding Parkinson’s neuropathology and its evolution through the nervous system. In addition, progress has been made towards understanding the molecular and neurophysiological mechanisms characteristic of this disease.

The present-day therapeutic strategy for alleviating Parkinson’s disease (PD) is to escalate dopamine levels in the brain. Therefore, Levodopa (L-DOPA) has remained the most vital drug for the management of PD symptoms for the last 30 years. However, the long-term use of L-DOPA is accompanied by some side effects, such as tardy action and a disabling dyskinesia termed “Levodopa-induced dyskinesia” [118]. Thus, innovative drugs and drug delivery systems are compulsory to increase drug efficacy and diminish side effects in PD patients.

Rotigotine represents a non-ergot derived D3/D2/D1 agonist and that has been stated to have neuroprotective properties and to lighten the motor symptoms of PD [119]. Xiuju Yan et al. [120] developed lactoferrin-modified rotigotine nanoplatforms (Lf-R-NPs) and studied their biodistribution, pharmacodynamics and neuroprotective effects, subsequent with nose-to-brain delivery in the 6-hydroxydopamine (6-OHDA) rat model of PD. It was shown that Lf-R-NPs rapidly passed in the brain and exhibited an improved sustained-release outcome compared with lactoferrin-free R-NPs. In addition, based on the performed pharmacodynamic study, Lf-R-NPs presented a comparatively higher efficacy in delivering rotigotine n the 6-OHDA PD rats. Additionally, Lf-R-NPs alleviated nigrostriatal dopaminergic neurodegeneration considerably.

Rasagiline (RSG) is a powerful inhibitor of monoamine oxidase type B (MAO-B) enzyme that deactivates biogenic amines (such as dopamine) in the CNS [121]. Therefore, the inhibition of MAO-B restores dopamine levels. A recent study assessed the improved brain targeting of RSG by using composite carriers based on poly(lactide-co-glicolide) nanoparticles (PLGA-NPs) coated with chitosan (CS). The aim of the study was to deliver and improve the bioavailability of RSG in brain tissue after administration of composite nano-formulations loaded with rasagiline (RSG-loaded-CS-coated-PLGA-NPs), as well as to reach an elevated therapeutic degree and, at the same time, to diminish the undesirable systemic exposure. The pharmacokinetic results of RSG-CS-PLGA-NPs after they were applied in a Wistar rat brain and plasma showed a high (** *p*  <  0.005) AUC_0–24_ (area under curve during 24 h) and an amplified C_max_ (maximum serum concentration) values over the intravenous treatment group. In vivo studies revealed the significance of intranasal administration and evidenced the beneficial used of the olfactory administration pathway for the effective treatment of Parkinson’s disease and related brain disorders by RSG-loaded-CS-coated-PLGA-NPs [122].

Neuropathologically, PD is described by a discriminating decrease of dopaminergic neurons mostly in the *substantia nigra pars compacta*, together with the formation of intracytoplasmic inclusions known as Lewy bodies, which stain positively for α-syn [123]. Genetic studies provided insights into the mutations of 6 genes (SNCA, Parkin, DJ-1, PINK1, LRKK2, and ATP13A2) that are the cause of familial PD. Over the last years, innovative gene therapies have been shown to be viable treatment options for PD. For example, a novel inner/outer magnetic NPs that carries shRNA plasmid to interfere with α-syn synthesis was developed. The authors proved that the NPs produced offer an operative repair in a PD model both in vitro and in vivo and further inhibited the apoptosis. As a result, α-syn expression was reduced, therefore averting the toxic effects of α-syn on the cell and defeating apoptosis [124]. Another group successfully synthesized composites based on gold NPs modified with chitosan (CTS), by which the plasmid DNA (pDNA) could be the load and specific cell types could be targeted by means of nerve growth factor (NGF) binding. The CTS@GNP-pDNA-NGF systems that resulted were transfected into cells by NGF receptor-mediated endocytosis, in order to suppress the expression of α-syn and inhibit the apoptosis of PC12 cells and *substantia nigra* striatum dopaminergic neurons. The data confirmed that CTS@GNP-pDNA-NGF presented notable therapeutic properties in both in vitro and in vivo PD models [125].

In another study, an amplified development of new neurons in the olfactory bulb of a mouse model with Parkinson’s disease has been achieved by an intracerebroventricular injection of microRNA-124-loaded NPs. Moreover, it was evidenced that microRNA-124-loaded NPs improved the relocation of newly established neurons into the lesioned striatum of mice and initiated an enhancement in motor functions [126].

## 4. Conclusions and Future Perspectives

When it comes to specific, selective and patient-oriented drug therapy, tremendous outcomes have been achieved and impressive possibilities reside in considering nanotechnology-derived materials and devices. In particular, designing nanoscale platforms for CNS conditions is even more challenging and intricate that conventional drug delivery. Besides the mandatory requirements (such as biocompatibility, biodegradability, biodistribution, accurate pharmacokinetics and pharmacodynamics, maximal therapeutic effects and minimal side effects), a nanostructured or nanosized system intended for CNS therapy must consider the particularity of brain tissue. The progress reported in molecular and cellular biology and the impressive advance of modern biomedicine enabled an intimate understanding of the CNS intrinsic barriers (with a particular emphasis on the BBB), which represent the innate protection structures of the human brain against exogenous and endogenous molecules (including antigenic and therapeutic ones).

In this framework, nanotechnology—mainly by means of nanoparticles—provides an attractive and modern alternative in order to develop innovative platforms for CNS conditions treatment. Thanks to their intrinsic features (mainly guided by size-related and surface-related properties), NPs represent ideal and versatile candidates for the engineering of performance-enhanced nanopharmaceuticals. As discussed within the previous sections, NPs have already been assessed as promising platforms for symptom management, neuroprotection or even disease regression in the case of AD and Parkinson’s conditions. Even if a certain treatment is not still available for chronic neurodegenerative diseases, the collaboration between healthcare researchers, materials scientists and engineers provides a beneficial and promising path towards the modern therapy desideratum.
